# Hematopoietic Stem Cell: Regulation and Nutritional Intervention

**DOI:** 10.3390/nu15112605

**Published:** 2023-06-01

**Authors:** Siyuan Sun, Yingxue Han, Yumei Lei, Yifei Yu, Yanbin Dong, Juan Chen

**Affiliations:** 1 Key Laboratory of Precision Nutrition and Food Quality, Department of Nutrition and Health, China Agricultural University, Beijing 100190, China; 2 National Engineering Laboratory for Animal Breeding, Key Laboratory of Animal Genetics and Breeding of the Ministry of Agriculture, College of Animal Science and Technology, China Agricultural University, Beijing 100193, China; 3 Key Laboratory of RNA Biology, Institute of Biophysics, Chinese Academy of Sciences, Beijing 100045, China

**Keywords:** hematopoietic stem cell, nutrients, RNA-binding protein, high-fat diet, bone marrow transplantation

## Abstract

Hematopoietic stem cells (HSCs) are crucial for the life maintenance of bio-organisms. However, the mechanism of HSC regulation is intricate. Studies have shown that there are various factors, either intrinsically or extrinsically, that shape the profile of HSCs. This review systematically summarizes the intrinsic factors (i.e., RNA-binding protein, modulators in epigenetics and enhancer–promotor-mediated transcription) that are reported to play a pivotal role in the function of HSCs, therapies for bone marrow transplantation, and the relationship between HSCs and autoimmune diseases. It also demonstrates the current studies on the effects of high-fat diets and nutrients (i.e., vitamins, amino acids, probiotics and prebiotics) on regulating HSCs, providing a deep insight into the future HSC research.

## 1. Introduction

Hematopoietic stem cells (HSCs) are multipotent precursors with the unique ability to self-renew into all cell types and self-regenerate in order to resume proliferation in the blood-forming system. These cells were first discovered in the bone marrow. Specifically, scientists discovered that a set of stem cells showed a hematopoietic function when they were intravenously injected into normal adult mice that had received a lethal dose of radiation [1]. The transplanted cells exhibited the ability to reestablish the destroyed hematopoietic system by differentiating into enough lymphoid and myeloid cells to support life [2]. The HSC niche is perivascular, can be created by mesenchymal stromal cells and endothelial cells and is likely to be located near the trabecular bone [3]. Human diseases related to HSCs encompass a broad spectrum of disorders that impact the normal functioning and development of the blood-forming system. These diseases arise due to abnormalities or dysregulation within the HSC population, leading to various pathological conditions. Examples of HSC-related diseases include hematological malignancies such as leukemia, in which the uncontrolled proliferation and differentiation of HSCs result in the accumulation of abnormal blood cells. Since the homeostasis of HSCs determines health in humans, understanding the intrinsic regulatory factors in HSCs and the extrinsic nutritional interventions needed for human health is important.

Investigation into the functioning of HSCs remains a prominent and dynamic area of exploration by researchers. In the past, studies on the internal factors regulating HSCs have mainly focused on transcription factors; however, in recent years, the focus has changed to encompass the regulatory role of RNA-binding protein and noncoding RNA families in HSCs. Nonetheless, reviews that summarize the role that nutrients play in HSC function are rare. Nutrients, including carbohydrates, lipids, proteins, vitamins and minerals, which are either energy sources for growth and reproduction or metabolic regulators, play pivotal roles in maintaining all forms of life. Generally, organisms utilize nutrients through two mechanisms: catabolic reactions and anabolic reactions. Nutrients with large molecular weights are broken down into small molecules, and through catabolic reactions, these molecules generate energy. In addition, small molecules are the basis of larger molecules that function via anabolic reactions. The integrated regulation of both processes enables the bioactivity that supports life. Few studies have elaborated on the connection between HSC function and dietary patterns, such as a high-fat diet [4,5,6,7,8]. Moreover, basic nutrients such as the vitamins ingested daily have also been proven to promote the function of HSCs [9,10,11,12,13].

In this review, we will describe the factors that affect HSC function and summarize the different components of nutrients that exert influences on HSCs. In the first part, we discuss mainly the intrinsic factors and regulatory mechanisms underlying HSC functions. In the second part, we outline recent findings showing how changes in an organism’s metabolic state induced by altered dietary nutrient intake may affect HSC homeostasis. A better understanding of the intrinsic factors regulating HSC homeostasis will help us determine the changes to these intrinsic factors induced by ingested nutrients, thus establishing a direct link between internal and external factors and ultimately improving human health by ensuring healthy HSC cell homeostasis.

## 2. Factors Regulating the Homeostatic Function of Hematopoietic Stem Cells

An increasing number of regulators have been reported to change HSC function. In general, these regulators can be categorized into several groups according to their functions. We discuss these groups in the following paragraphs.

### 2.1. RNA-Binding Protein

**Figure 1:**
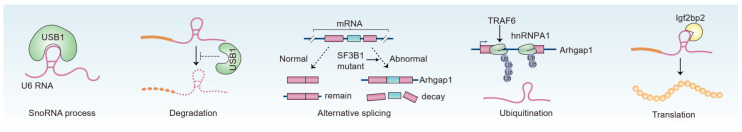
Different mechanisms of RNA-binding protein interacting with its target RNA. U6 biogenesis 1 (USB1) excises U6 and U6atac small nuclear RNAs, and USB1 deadenylates miRNAs to avoid their degradation. Wrong splicing of *SF3B1* mutants results in nonsense-mediated decay of RNA. TRAF6 ubiquitinates of hnRNPA1, causing abnormal alternative splicing of Arhgap1. Igf2bp2 regulates translation of mRNA through Lin28b/Hmga2 pathway.

RNA-binding proteins bind specific RNAs to manipulate RNA bioactivity, which in turn regulates cell function. An increasing number of studies have revealed the regulatory functions of RNA-binding proteins in HSCs (Figure 1).

As one type of RNA-binding protein, splicing factors participate intron removal from mRNA, which enables proteins to be accurately translated. Four genes (*SF3B1*, *SRSF2*, *U2AF1* and *ZRSR2*) encode splicing factors, and mutations in these genes are frequently reported in leukemia [14,15,16,17]. For example, a common mutation in *SF3B1* (K700E) contributes to the expansion of long-term HSCs (LT-HSCs) and the phenotype acquisition of myelodysplastic syndrome (MDS). More than 80% of patients with myelodysplastic syndrome with ring sideroblasts (MDS-RS) carry mutations in the *SF3B1* gene; during bone marrow transplantation, *SF3B1*-mutated MDS-RS HSCs differentiate into characteristic ring sideroblasts [18]. One study demonstrated that the K700E mutation contributed to abnormal 3′ splice-site selection and increased the nonsense-mediated decay of RNA [19]. Lieu et al. showed that this mutation promotes the mis-splicing of MAP3K7 and ultimately accelerates the death of erythrocytes and leads to the acquisition of the MDS phenotype [20]. In addition, mutations in *SF3B1* and *SRSF2* exert convergent effects by enhancing autophagy in hematopoietic stem and progenitor cells (HSPCs) and increasing the cell death rate through the hyperactivation of NF-κB signaling [21]. A study reported that heterozygous mice carrying the *SRSF2* P95H mutation exhibit a significant reduction in the number of HSPCs and an increased number of HSPC differentiation defects under both steady-state conditions and after transplantation [22]. Fang et al. reported that hnRNPA1, an auxiliary splicing factor, is a substrate of TRAF6. Ubiquitination of hnRNPA1 by TRAF6 regulates the alternative splicing of Arhgap1, which is critical for the hematopoietic defects observed in TRAF6-expressing HSPCs [23].

In addition to these splicing factors, other RNA-binding proteins have also been reported to regulate the activity of HSCs. U6 biogenesis 1 (USB1) was discovered to be a 3′→5′ exoribonuclease that removes 3′-terminal uridine bases from U6 small nuclear RNA transcripts. In addition, USB1 has been recently reported to play a critical role in deadenylating microRNAs (miRNAs) and retarding their degradation, and mutations in USB1 can affect miRNA levels during blood development by inhibiting the removal of 3′-end adenylated tails, which impairs hematopoietic development [24]. Igf2bp2, which is located downstream of the Lin28b/Hmga2 pathway, is an RNA-binding protein that regulates the stability and translation of mRNA. A recent study showed that Igf2bp2-dependent gene regulation in young HSCs and a decline in its function in aged HSCs both contributed to the acquisition of distinct phenotypes associated with HSC aging [25]. Musashi 2 (Msi2), a translational inhibitor, maintains the stem cell compartment mainly by regulating the proliferation of primitive progenitors downstream in LT-HSCs. Through cell cycle and gene expression analyses, the decreased proliferation capacity of ST-HSCs and lymphoid myeloid-primed progenitors (LMPPs) was discovered in Msi2-defective mice. Moreover, HSCs with Msi2 knocked out exhibited significant defects in competitive repopulation experiments [26]. By overexpressing MSI2, one study showed that pro-self-renewal phenotypes of HSCs can be induced, resulting in a 17-fold increase in the number of ST-HSCs and a 23-fold increase in the number of LT-HSCs. Furthermore, in a comprehensive analysis of MSI2-RNA interactions, MSI2 was shown to dampen aryl hydrocarbon receptor (AHR) signaling directly in cord blood HSPCs. This regulatory effect was mediated through the posttranscriptional downregulation of key components involved in the canonical AHR pathway [27]. Zfp36l2 is a critical modulator of definitive hematopoiesis. Zfp36l2-KO mice showed a significant reduction in the levels of both definitive multilineage and hematopoietic progenitors, suggesting that Zfp36l2 functions as an essential target to control the stability of mRNA in HSPCs [28].

Taken together, these observations identify a class of RNA-binding proteins that maintain the self-renewal and differentiation of HSCs, and these RNA-binding proteins regulate the self-renewal and differentiation of HSC by regulating RNA transcription, splicing and translation. These intrinsic regulatory mechanisms provide a rich molecular basis for later nutritional interventions to extend HSC homeostasis.

### 2.2. Epigenetic Regulation of HSC

Several components of the m6A machinery, including METTL3, ALKBH5, a m6A eraser α-ketoglutarate-dependent dioxygenase (FTO) and the m6A reader YTH N6-methyladenosine RNA-binding protein 2 (YTHDF2), are commonly dysregulated in leukemia [29,30,31,32]. It has been reported that the m6A writer enzyme METTL3 is highly expressed in acute myeloid leukemia (AML) cells, and its loss promotes cell differentiation and decreases cell proliferation in HSPCs [30]. One study showed that either the downregulation of YTHDF2 or the activation of FTO inhibited *CXCR4* decay in cord blood (CB) HSCs, promoting their homing and engraftment activity [33]. YTHDF2 is an m6A reader that recognizes m6A-modified transcripts and participates in m6A-mRNA degradation. Recent studies have revealed that YTHDF2 functions as a repressor of inflammatory pathways in HSCs and plays an essential role in long-term HSC maintenance [34,35]. Moreover, its inactivation leads to HSC expansion and ameliorates AML, while YTHDF2 deficiency exerts no effect on normal HSC function [31]. These results suggest a potential strategy to reduce leukemic stem cell (LSC) survival.

In vivo experiments showed that m6A demethylation by ALKBH5 fine-tunes the activity of splicing factor encoded by *SF3B1* and other epigenetic regulators, indicating that ALKBH5 may delay HSC leukemic transformation in patients with MDS [36]. STED2, a histone H3 lysine 36 methyl-transferase, plays an important role in the pathogenesis of hematologic malignancies. A recent study showed that STED2 deficiency facilitated the self-renewal of NHD13^+^ HSPCs, which promoted the transformation of MDS into AML [37]. The Ψ writer PUS7 plays a dual role by modifying and activating a network of tRNA-derived small fragments (tRFs) that target the translation initiation complex. When PUS7 is inactivated in embryonic stem cells, the translational regulation mediated by tRFs is impaired, resulting in increased protein biosynthesis. Thus, the disruption of the posttranscriptional regulatory mechanism leads to a reduced commitment of hematopoietic stem cells and an increased susceptibility to MDS development in humans [38].

Highly expressed in adult HSCs, MLLT3 (also known as AF9) is a component of super elongation complex 6 and interacts with DOT1L to exert its effects. MLLT3 sustains the abundance of H3K79me2 and is an HSC maintenance factor that connects histone readers with other modification factors to regulate the expression of HSC-specific genes. Moreover, in a mouse model, the sustained expression of MLLT3 led to balanced multilineage reconstitution in both the primary and secondary recipients [39].

**Figure 2:**
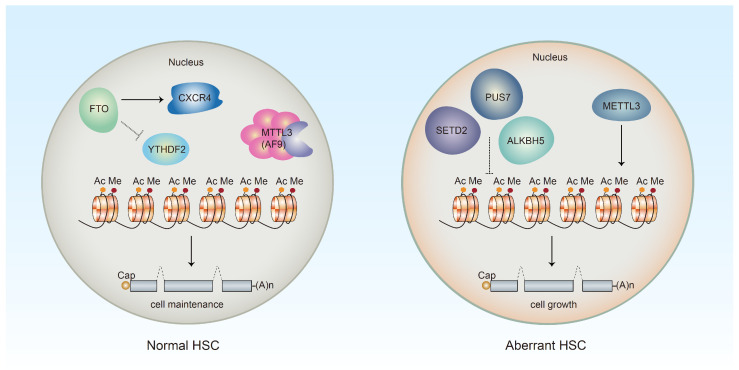
Factors involved in the regulation of different states of HSCs in epigenetics. In normal HSCs, YTHDF2, the m6A reader, maintains long-term HSCs, while α-ketoglutarate-dependent dioxygenase (FTO) acts against YTHDF2, inhibiting *CXCR4* decay to promote homing and engraftment activity of HSCs. In addition, MLLT3 sustains H3K79me2 level to maintain HSCs. In the pathogenesis of hematopoietic malignancies, METTL3 increases the HSPC growth in acute myeloid leukemia (AML). ALKBH5 delays HSC leukemic transformation in myelodysplastic syndromes (MDS), and STED2 deficiency facilitates the transformation of MDS into acute myeloid leukemia (AML). Moreover, inactivation of the Ψ “writer” PUS7 also contributes to the impairment of HSCs and the development of MDS.

These epigenetic regulators regulate the division pattern of HSCs, which subsequently controls the self-renewal and differentiation of HSCs (Figure 2), thus maintaining the normal development and homeostasis of the hematopoietic system.

### 2.3. Other Factors Regulating HSC Dormancy and Maintenance

Generally, cells in organisms must strike a balance between self-renewal and differentiation. In a stable environment, most HSCs are in a state of quiescence, as frequent differentiation of HSCs reduces the HSC pool. HSCs maintain dormancy through the action of multiple transcription factors. SIRT7 facilitates the transcription of DNA, and its expression is reduced in aged HSCs. Researchers found that its inactivation reduced the number of HSCs in a quiescent state and compromised HSC regenerative capacity. SIRT7 upregulation increased the regenerative capacity of aged HSCs [40]. Hu et al. demonstrated that steroid receptor coactivator 3 (SRC-3) was highly expressed in HSCs. *SRC-3*^−/−^ mice were used to measure the function of SRC-3, and it was revealed that SRC-3 maintained the quiescence of HSCs and removed ROS from mitochondria [41]. Adenosine-to-inosine RNA editing and the enzyme adenosine deaminase play crucial roles in hematopoietic cell development and differentiation. Specifically, antizyme inhibitor 1 (Azin1) has been observed to undergo extensive editing in hematopoietic stem and progenitor cells (HSPCs). This editing process leads to an amino acid change, resulting in the translocation of the Azin1 protein (AZI) to the nucleus. In the nucleus, AZI exhibits an enhanced binding affinity for DEAD box polypeptide 1 and modulates the expression of multiple hematopoietic regulators, ultimately promoting HSPC differentiation [42].

Zpf70 is a transcription factor that regulates the self-renewal and differentiation of hematopoietic stem cells. By using CRISPR/Cas9 technology to delete Zfp90, researchers found that Zpf70 promotes HSC self-renewal in a Hoxa9-dependent manner. Specifically, Zfp90 binds to the promoter of Hoxa9 to initiate its expression via the NURF complex [43]. Autophagy suppresses the metabolism of hematopoietic stem cells by selectively eliminating functional and healthy mitochondria. This process is crucial for maintaining the quiescent state and stemness of cells. Additionally, autophagy becomes increasingly important with age, as it preserves the regenerative capacity of aging hematopoietic stem cells [44]. HSCs with a high receptor tyrosine kinase Tie2 expression tend to be quiescent and resist undergoing apoptosis. The binding of Tie2 with its ligand angiopoietin-1 (Ang-1) maintains the long-term repopulation of HSCs in vivo [45]. The activation of mitophagy, a quality control mechanism in mitochondria, is crucial for the self-renewal and expansion of Tie2^+^ hematopoietic stem cells (HSCs). The PPAR (peroxisome proliferator-activated receptor)-fatty acid oxidation pathway actively promotes the expansion of Tie2^+^ HSCs by facilitating the recruitment of Parkin to mitochondria [46]. One study reported that the activity of chaperone-mediated autophagy (CMA) in HSCs decreased with age and showed that the genetic or pharmacological activation of CMA restored the functionality (lower intracellular ROS levels, increased PK and GAPDH activities, etc.) of HSCs in both old mice and elderly humans [47]. These findings focused on the regulatory mechanisms of HSCs mediated by enhancer–promoter interactions and provided a theoretical basis for the future exploration of HSCs in this spatial field, leading to greater resolution of the multidimensional genetic regulatory mechanisms underlying HSC production.

### 2.4. Phase Separation

**Figure 3:**
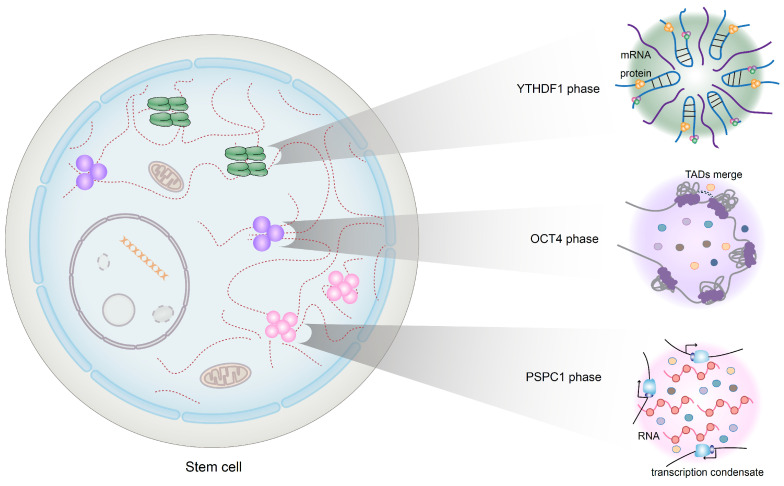
Several proteins participating in the formation of phase separation in stem cells. YTHDF1 interacts with IKBα/β mRNAs to form phase separation, which ultimately promotes the transformation from spermatogonial stem cells (SSC) to induced neural stem-cell-like cells (iNSC). Phase separation of OCT4 reorganizes the structure of topological-associated domains (TADs), which manipulates induced pluripotent stem cell (iPSC) generation. PSPC1 binds RNA and forms transcription condensates, increasing transcription process in embryonic stem cells (ESC).

Phase separation has recently become a hot topic since a consensus has been reached suggesting that biomolecular condensates with membranes are reaction platforms for biological functions [48]. Limited research on the influence of phase separation on HSCs has been reported, and the mechanisms established are shown in Figure 3. One study showed that the transdifferentiation of spermatogonial stem cells (SSCs) into induced neural stem-cell-like cells (iNSCs) is mediated by the methylation reader protein YTHDF1 interacting with IKBα/β mRNAs, which promotes the formation of phase-separated condensates. The formation of condensates inhibits the translation of IKBα/β mRNAs and activates nuclear factor κB (NF-κB) p65, which ultimately facilitates the transdifferentiation of SSCs mediated through the expression of Eya1 [49]. Shao et al. revealed a mechanism in embryonic stem cells (ESCs), in which promoter-associated RNAs and their binding proteins cooperatively initiate the phase separation of polymerase condensates to promote transcription. Specifically, the paraspeckle protein PSPC1 combines with RNA, which functions as a multivalent molecule. This interaction results in the formation of transcript condensates and leads to the subsequent phosphorylation and release of RNA polymerase (Pol) II, increasing the activity of the polymerase [50].

During the development of acute promyelocytic leukemia (APL), the aberrant phase separation of PML/RARα caused by neddylation of the RARα moiety results in the failure of PML nuclear body (NB) assembly, which is essential for tumor suppression. Moreover, when PML/RARα was deneddylated, phase separation was reinitiated and functional NBs were formed, and PML/RARα-driven leukemogenesis was also impeded [51]. Phase separation has been found to influence epigenetic modifications in cancer stem cells (CSCs), specifically regulating their self-renewal activity. It has been hypothesized that phase separation promotes the tumorigenicity of CSCs through ubiquitination. For example, the mutation of speckle-type POZ protein (SPOP), which prevents the recruitment of ligase substrates, facilitates the accumulation of proto-oncoproteins and triggers the phase separation of SPOP, maintaining the ubiquitination of ubiquitin-dependent proteins [52]. OCT4 is known as a master transcription factor for somatic cell reprogramming. Wang et al. recently demonstrated that the phase separation of OCT4 contributes to topological-associated domain (TAD) reorganization, which affects the efficiency of induced pluripotent stem cell (iPSC) generation [53].

The dysregulation of protein homeostasis plays a pivotal role in the aging process of hematopoietic stem cells (HSCs). In a recent study, researchers examined the proteome of HSCs and identified crucial protease inhibitors. Their findings revealed that the genetic elimination of prolyl isomerase resulted in the accelerated aging of HSCs. Interestingly, prolyl isomerase facilitates phase separation, thereby enhancing cellular stress resistance. These discoveries highlight the significance of maintaining the protein balance in HSCs and shed light on the role of prolyl isomerase in regulating HSC aging [54]. This study linked a family of widely expressed chaperones to phase transitions and revealed that macromolecular condensation dynamics drive the aging of blood stem cells.

## 3. Therapies for Bone Marrow Transplantation

The homing and engraftment of hematopoietic stem cells are crucial for the efficiency of bone marrow transplantation, and when donor-cell numbers are low, clinical engraftment can be severely compromised. Christopherson et al. reported that endogenous CD26 expression on donor cells negatively regulates homing and engraftment. The inhibition or deletion of CD26 greatly increases the efficiency of transplantation [55]. A group of researchers showed that the administration of ACK2, an antibody that inhibits c-kit function, resulted in the temporary depletion of more than 98% of endogenous hematopoietic stem cells (HSCs) in immunodeficient mice. Subsequent transplantation of donor HSCs into these mice led to high levels of chimerism, which was as high as 90%. Extrapolating these findings to humans may potentially facilitate the development of mild yet effective conditioning regimens for transplantation [56]. One finding revealed that in competitive repopulation experiments, hemizygous *CXCR4* (*Cxcr4*^+/o^) HSCs presented a stable proliferation without depleting long-term hematopoietic stem cells (LT-HSCs), indicating that partial inactivation of *CXCR4* may be a strategy to promote HSC engraftment in patients who have WHIM syndrome (*Cxcr4^+/S338X^*) after transplantation [57]. UM171 functions by augmenting the self-renewal capacity of human LT-HSCs regardless of whether AhR is suppressed. In contrast, the activity of AhR inhibitors seems to be limited to cells with transient self-renewal capacity. In the in vitro expansion of LT-HSCs and cells derived from them, using UM171 may be an alternative approach for prioritizing small, well-HLA-matched cord blood units as potential cell sources for transplantation involving donor selection algorithms in the future [58]. Moreover, viral therapy has recently been used to cure specific types of HSC-related diseases.

Since matched donors are not always available, the infusion of autologous HSPCs modified ex vivo via gene therapy is considered to be an alternative approach to hematopoietic stem/progenitor cell (HSPC) transplantation in the treatment of many diseases. Researchers used a lentiviral vector encoding functional WASP, a protein regulating the cytoskeleton, to genetically correct HSPCs obtained from three Wiskott–Aldrich syndrome (WAS) patients and reinfused the edited cells after a reduced-intensity conditioning regimen was administered [59]. Cartier et al. initiated the lentiviral-mediated gene therapy of hematopoietic stem cells in X-linked adrenoleukodystrophy (ALD) patients and found that this therapy led to successful allogeneic hematopoietic cell transplantation, indicating that this approach offered therapeutic benefits to ALD patients [60]. Metachromatic leukodystrophy (MLD), usually caused by arylsulfatase A (ARSA) deficiency, is an inherited lysosomal storage disease. Researchers used a lentiviral vector to transfer a functional ARSA gene into hematopoietic stem cells (HSCs). After the reinfusion of the gene-corrected HSCs, extensive and stable ARSA gene replacement was detected in MLD patients, suggesting a new method to cure MLD [61].

## 4. The Relationship between HSCs and Autoimmune Diseases (ADs)

ADs are characterized by the immune system attacking the host’s own tissues due to the loss of self-tolerance [62], and HSCs have been implicated in the pathogenesis of autoimmune diseases [63,64,65]. They are involved in the production of lymphocytes, specifically T cells and B cells, which play a central role in autoimmune responses. Abnormalities in the differentiation or regulation of lymphocytes derived from HSCs can lead to the production of self-reactive cells, contributing to the development of autoimmune diseases. It has been reported that CD8 T cells are detrimental to BM failure and the function of HSCs, causing anemia [63]. In one study, it was found that HSCs of patients with type 1 diabetes were possibly genetically programmed to facilitate the proliferation of autoreactive memory B cells [64]. Another study showed that the hyperactivation of mTOR impaired the hematopoiesis of HSCs in mice [65].

Hematopoietic stem cell transplantation (HSCT) holds its potential as a therapeutic approach to autoimmune diseases (ADs). Studies demonstrated that HSCT could serve as an effective treatment in some severe ADs such as multiple sclerosis, systemic sclerosis and systemic lupus erythematosus [66,67]. Manipulating HSCs to enhance immune tolerance or modulate the inflammatory microenvironment may offer promising avenues for future treatment strategies.

## 5. High-Fat Diet (HFD) Affects the Activity of HSCs

The mTORC1 pathway tends to be elevated in overnutrition conditions such as obesity, which can subsequently induce diabetes [68]. It has been reported that during nutrient deprivation, two repressors of mTORC1, seizure threshold 2 (SZT2) and tuberous sclerosis complex-1 (TSC1), are crucial for HSC homeostasis. The loss of SZT2 in HSCs decreased the HSC pool and reduced the repopulating capacity of HSCs. Moreover, the ablation of both SZT2 and TSC1 markedly depleted the HSC pool and led to a marked synergistic effect that increased mTORC1 activity and ROS production by 10-fold [4].

Spred1, which is a negative regulator of RAS-MAPK signaling, has been reported to participate in maintaining HSC homeostasis, particularly in animals fed on HFDs. Specifically, under normal conditions, Spred1 negatively modulates HSC self-renewal and fitness, partially through the regulation of Rho kinase activity. Spred1 deficiency mitigates HSC failure induced by pathogen mimetics, extending the HSC lifespan, but it does not initiate leukemia cells due to the compensatory upregulation of Spred2. However, after animals were fed on an HFD, Spred1-deficient HSCs exhibited a hyperactivation of the ERK pathway and abnormal self-renewal, resulting in functional HSC failure, severe anemia and the development of myeloproliferative neoplasm-like disease. These HFD-induced hematopoietic abnormalities were, at least in part, mediated by alterations in the gut microbiota [5].

Hermetet et al. investigated the impact of HFDs on TGF-β signaling in HSCs [6]. They utilized C57BL/6J model mice fed on an HFD and a standard diet in a short-term study and observed a reduction in the number of primitive HSCs among the BM cells of the HFD-fed mice. These HFD-fed mice exhibited a diminished hematopoietic reconstitution potential after transplantation. The disrupted maintenance of HSCs was attributed to a reduction in the number of dormant HSCs following an HFD intake. The researchers also found that mice fed on an HFD showed disrupted TGF-β receptor localization within lipid rafts, leading to impaired Smad2/3-dependent TGF-β signaling, which was the primary molecular mechanism underlying these effects. However, the administration of recombinant TGF-β1 to HFD-fed mice attenuated the loss of HSCs and the compromised recovery ability of the BM cells. Another study revealed that metabolic stress induced by HFDs affected the bone marrow niche by altering the gut microbiota and the balance between the numbers of osteoblasts and adipocytes. Specifically, HFDs resulted in a decrease in the number of long-term Lin^−^ Sca-1^+^ c-Kit^+^ (LSK) stem cells and a shift in the number of cells differentiating from lymphoid to myeloid lineages. The function of the bone marrow niche was impaired after HFD intake, as shown by the poor reconstitution of hematopoietic stem cells. HFD feeding led to the robust activation of PPARγ2, which hindered osteoblastogenesis while promoting adipogenesis in the bone marrow. Additionally, the expression of key genes, such as Jag-1, SDF-1 and IL-7, involved in forming the bone marrow niche was significantly suppressed after HFD intake. Moreover, HFD-feeding-induced changes in the structure of the gut microbiota were associated with alterations in the bone marrow. The partial rescue of HFD-mediated effects on the bone marrow niche was achieved through antibiotic treatment, and the transplantation of stools from HFD-fed mice into normal mice led to the normal mice presenting with the same symptoms as the HFD-fed mice [7].

Interestingly, the HSC pattern was passed to the next generation. Maternal obesity, especially when combined with an HFD intake during pregnancy, has been found to impede the normal expansion of fetal HSPCs while promoting the differentiation of these cells into other cell types. Notably, these effects were only partially attenuated when dietary adjustments were made for mothers with obesity during pregnancy. Through competitive transplantation experiments, HSPCs were shown to be programmed by HFDs and exhibited a reduced ability to repopulate the hematopoietic system and showed a bias toward myeloid cell differentiation. Importantly, these impairments were found to depend on the microenvironment in HFD-conditioned male recipients. Deficiencies in fetal HSPCs coincided with disruptions in the expression of genes involved in metabolism, immune and inflammatory processes and the stress response. Additionally, genes that are crucial for hematopoietic stem cell self-renewal were downregulated, and the activation of pathways that regulate cell migration was inhibited [8]. Another study demonstrated that in type 2 diabetic HFD-fed mice, elevated blood sugar levels, the criteria for a hyperglycemia diagnosis, led to the simultaneous expression of insulin and TNF-α in Lin^−^ Sca-1^+^ c-Kit^+^ (LSK)progenitor cells. This coexpression pattern was maintained in the progeny of these cells. Interestingly, when these progenitor cells were transferred to animals with normal blood sugar levels, they were found fuse with neurons [69].

## 6. Specific Nutrients Essential to Maintain HSC

As an indispensable group of substances to support life, nutrients can affect HSCs in many aspects. Here we discuss how vitamins, amino acids, prebiotics and probiotics exert their influences on the behavior of HSCs and HSC-related diseases (Table 1).

### 6.1. Vitamins

#### 6.1.1. Vitamin A

Targeting vitamin A receptors may be a therapy for chronic graft-versus-host disease (GVHD). Heat shock protein 47 (HSP47) contributes to multiorgan fibrosis during allogeneic HSCT and reduces the life expectancy of patients after SCT. Through treatment with HSP47 small interfering RNA (siRNA) delivered via vitamin-A-coupled liposomes, HSP47 expression was downregulated in cells expressing vitamin A receptors, which effectively attenuated fibrosis [70]. The serum concentrations of retinol-binding protein and transferrin are considered to be biochemical indices for the assessment of individual nutritional status during autologous and allogeneic HSCT [9]. The stimulation of retinoic acid (RA) signaling in HE (aorta-gonad-mesonephros-derived hematopoietic endothelial) cells in an ex vivo setting significantly enhanced their potential to become HSCs. In contrast, when the enzyme critical for RA metabolism, retinal dehydrogenase 2, was conditionally inactivated in VE-cadherin-expressing endothelial cells in vivo, HSC development was completely abolished. Wnt signaling completely inhibited the HSC-inducing effects of RA modulators. In contrast, inhibiting the Wnt pathway promoted HSC development even in the absence of RA signaling [10]. Moreover, treatment with all-trans retinoic acid hindered the activation of HSCs induced via stress through the mechanism involved in protein translation suppression and reactive oxygen species (ROS) and Myc level reduction. Mice that were maintained on a vitamin-A-free diet experienced a loss of HSCs and exhibited disrupted HSC reentry into a dormant state following exposure to inflammatory stress stimuli [11].

#### 6.1.2. Vitamin B3

In a mouse model, dietary supplementation with vitamin B3, an NAD^+^ precursor nicotinamide riboside, increased HSC function by stimulating hematopoiesis. NAD^+^ is a vital coenzyme in many fundamental reactions, such as those in the TCA cycle, that generates energy, and it has also been found to increase mitophagy and reduce mitochondrial metabolism rates [71]. A recent study revealed that dietary supplementation with NAD^+^ precursors contributed to HSC function maintenance by attenuating mitochondrial stress and shrinking the mitochondrial network size, reducing the acquisition of the age-associated phenotype of HSCs [12].

#### 6.1.3. Vitamin C

Vitamin C, also known as ascorbate, is a common reductant that reduces ROS levels. Patients with low levels of vitamin C in plasma, which are possibly associated with ASXL1 mutations, are at greater risk for developing acute myeloid leukemia than other myeloid malignancies. One study reported that oral vitamin C supplementation temporarily restores and maintains adequate vitamin C concentrations (1 g twice daily) in patients undergoing myeloablative chemotherapy and stem cell transplantation. However, intolerance issues were reported in patients one week after chemotherapy treatment [72]. Thus, intravenous vitamin C may be a better treatment choice. Agathocleous et al. showed that systemic ascorbate depletion in mice increased HSC frequency and function because the effect was enhanced by Flt3 internal tandem duplication (Flt3^ITD^) leukemic mutations, while dietary ascorbate reversed the acceleration of leukemogenesis [13].

#### 6.1.4. Vitamin D

Rats were fed for one month without vitamin D or vitamin D supplementation, after which rat serum was collected, and bone marrow stem cells were cultured. The results showed that the stem cells divided at a slower rate and produced fewer stem cells than those in the group that received supplemental vitamin D. This study showed that targeted nutritional supplementation can restore the function of aging stem cells involved in the hematopoietic system [73]. One study demonstrated the effect of vitamin D on brain stem cells in a murine model of multiple sclerosis (MS). The investigators used an MS mouse model to determine whether vitamin D treatment increased neural function by protecting neuronal stem cells and promoting their function. Surprisingly, they found that vitamin D supplementation reversed the nerve cell damage caused by MS. This study provides additional insights into how vitamin D directly contributes to the mitigation of diseases caused by impaired stem cell function in adults [75]. In one study, subjects were provided supplements containing ellagic acid and vitamin D and fermented lactic acid from probiotic bacteria twice per day for 2 weeks. The researchers found a significant increase in the number of circulating bone marrow stem cells. This finding suggests that nutrients can enhance the function of stem cells and further delay the aging associated with stem cells [74].

### 6.2. Amino Acids

Restricting dietary valine in mice resulted in the depletion of the bone marrow niche and facilitated the engraftment of donor HSCs without the need for chemotherapy or irradiation. This suggests that valine plays a crucial role in maintaining HSC function, and dietary valine restriction may potentially reduce complications associated with HSC transplantation [76]. By using HSCs obtained from mice in ex vivo experiments, Wilkinson et al. [77] found that a branched-chain amino acid (BCAA) imbalance reduced HSC proliferation and survival, whereas low valine levels resulted in poor HSC maintenance. Recently, Li et al. discovered direct links between metabolic alterations and translation regulation in HSCs during homeostasis and proliferation. Specifically, they found that to maintain homeostasis, HSCs actively engaged in high-amino-acid (AA) catabolism to decrease cellular AA levels, and this effect was facilitated by activation of the GCN2-eIF2α axis. This process was a protein synthesis inhibitory checkpoint, limiting protein synthesis to support HSC maintenance. In this study, under proliferation-promoting conditions, HSCs exhibited enhanced mitochondrial oxidative phosphorylation (OXPHOS) and generated higher energy levels. This metabolic adaptation resulted in decreased AA catabolism and the accumulation of cellular AAs. As a consequence, the GCN2-eIF2α axis, which is critical for inhibiting protein synthesis, was inactivated. This inactivation led to increased protein synthesis and, in combination with proteotoxic stress, affected cellular proteostasis. GCN2 plays a pivotal role in the maintenance of proteostasis and the inhibition of Src-mediated AKT activation via this process to repress mitochondrial OXPHOS in HSCs. Therefore, GCN2 deletion reduces HSC repopulation and regeneration. Furthermore, the glycolytic metabolite nicotinamide riboside (NR), which is a precursor to NAD^+^, promotes the catabolism of AA to activate GCN2 and thus supports the long-term functionality of HSCs [78].

### 6.3. Probiotics and Prebiotics

Several reviews have noted that the administration of probiotics and prebiotics alleviates gut microbial dysbiosis in the contexts of acute leukemia, multiple myeloma and post-transplantation [79,80,81]. We summarize the recent studies on probiotics and prebiotics that indicated that they attenuated other disease symptoms in this section. Probiotics and prebiotics can alleviate the gastrointestinal symptoms caused by chemotherapy and autologous hematopoietic stem cell transplantation (auto-HSCT). Graft-versus-host disease (GVHD) occurs when alloreactive donor T cells attack the mucous layer, and it causes gut inflammation and bacterial translocation after allogeneic hematopoietic stem cell transplantation (allo-HSCT) treatment [82]. One finding revealed that prebiotic intake may be an alternative strategy for preventing acute graft-versus-host disease (aGVHD) after allo-HSCT. Specifically, prebiotic treatment attenuated mucosal injury and decreased the cumulative incidence of skin aGVHD. Concomitantly, the gut environment in the patients was effectively maintained, and the butyrate-producing bacterial population was preserved. Thus, prebiotic intake can be utilized to increase treatment outcomes after stem cell transplantation [83]. Synbiotics, a mixture of live microorganisms and substrate (s) selectively utilized by host microorganisms confers a health benefit on the host [84], and it reportedly ameliorates chemotherapy-induced mucosal damage. One RCT experiment demonstrated that the intake of *Bifidobacterium longum* (BB536) and guar gum significantly attenuated diarrhea and anorexia after engraftment, indicating that synbiotics attenuated gastrointestinal toxicity effects [85]. Although many studies have reported the beneficial effects of prebiotics and probiotics on bone marrow transplantation, the direct influence of prebiotics and probiotics on HSCs needs to be elucidated.

## 7. Future Perspectives

The homeostatic regulation of HSCs has been intensively studied in the past few decades. Most studies have focused on how transcription factors bind promoter or cofactors to regulate transcription and RNA-binding protein or noncoding RNA regulation via posttranscriptional or translational modifications, and affecting the homeostasis of hematopoietic stem cells is relatively preliminarily. How epigenetic regulation precisely controls hematopoietic stem cell function is unclear. Moreover, enhancer–promoter interactions and their regulatory networks may be important mechanisms contributing to HSC homeostasis. The chaperone properties of prolyl isomerase in HSCs suggest that condensates formed by biomacromolecules may play important roles in the renewal and self-differentiation of HSCs. Thus, an in-depth understanding of how RNA-binding proteins as well as epigenetic modifications regulate stem cell function will undoubtedly generate new strategies for the maintenance of healthy HSCs in vitro and in vivo. Understanding the intrinsic factors affecting HSCs will help us develop targeted nutritional interventions that will ultimately lead to dietary improvements in human health.

In this review, we introduced the definition of HSCs and summarized the factors that regulate HSC bioactivity. We also discussed various nutrients that can affect HSC activity, either directly or indirectly. However, there is limited research that can clearly demonstrate the interaction between specific nutrients and HSC activity. Current studies have mainly focused on identifying factors that directly regulate HSCs without exploring the links between these factors and nutrients. Clinical studies should be carried out more broadly and precisely. Most studies were based on mouse models, which ought to be applied further into human patients in a safe way. More methods have been developed to study HSCs. Adam and his colleagues created a new method to achieve the albumin-free and long-term ex vivo expansion of functional HSCs. Since serum albumin was one poorly defined albumin supplement in HSC cultures, this study made a fully defined culture by replacing serum albumin with polyvinyl alcohol [86].

In one study, a metabolomics method was utilized for the analysis of rare cell populations isolated directly from tissues and to compare mouse hematopoietic stem cells (HSCs) to restricted hematopoietic progenitors [13]. An article adopted single-cell RNA sequencing coupled with iterative clustering and guide-gene selection and clonogenic assays to identify different states of cells, which can be utilized to manifest genes of HSPCs from mixed-lineage intermediates [87]. Based on the role that nutrients play in HSCs, efficient therapies can be developed for patients possessing HSC-related disease, especially for those treated by bone marrow transplantation. One study showed that pretreatment with NAC (N-acetyl-L-cysteine) enhanced the engraftment of transplanted hematopoietic stem cells (HSCs) in the bone marrow [88]. This pretreatment facilitates the successful homing of HSCs to the bone marrow and may create a supportive microenvironment with low levels of reactive oxygen species (ROS) that helps in the retention of stem cells. These findings suggest that NAC pretreatment holds promise in improving HSC transplantation outcomes and maintaining a favorable microenvironment for HSC function.

Due to the complex digestion and absorption process of nutrients, it is necessary to track their transforming metabolism in the body and to identify the main products using biochemical techniques. Moreover, identifying the regulation of HSC homeostasis by these products could help us develop new foods that are healthier and safer. The molecular mechanisms of how these in vivo transformation products affect the genome, transcriptome or proteome of HSCs often involve multiple steps and therefore need to be explored through molecular networks using more advanced frontier technologies. As people around the world become increasingly concerned about the steady growth of their daily diet and life expectancy, it is still worthwhile to explore the link between daily nutrient intake and HSC function.

## Figures and Tables

**Table 1:** Effects of different nutrients on the influence of HSC.

Nutrients	Functional Changes	Regulatory Mechanism	Ref.
Vitamin A	Improves fibrosis, facilitates HSC development	Downregulates HSP47 expression, suppresses level of ROS	[55,56,57,58]
Vitamin B3	Stimulates hematopoiesis, attenuates age-associated metabolic and functional changes in HSC	A precursor to NAD^+^; increases mitophagy and reduces mitochondrial metabolism	[59,60]
Vitamin C	Improves acute myeloid leukemia condition, slackens leukemogenesis	Removes ROS, combines with Flt3 internal tandem duplication (Flt3ITD)	[61,62]
Vitamin D	Rescues aging stem cells, improves neural function	-	[63,64,65]
Amino acids	Maintains HSCs, reduces iatrogenic complications in HSC transplantation	Activates the GCN2-eIF2α axis, inhibits Src-mediated AKT activation	[66,67,68]
Probiotics, prebiotics and synbiotics	Prevents acute graft-versus-host disease (aGVHD), improves mucosal injury, ameliorates chemotherapy-induced mucosal damage improve diarrhea and anorexia after engraftment	Maintains butyrate-producing bacterial population, reduces gastrointestinal toxicity	[69,70,71,72,73,74]

## Data Availability

Not applicable.
